# The utilisation of procedural sedation and analgesia at a regional hospital emergency department in KwaZulu-Natal

**DOI:** 10.4102/jcmsa.v4i1.429

**Published:** 2026-07-31

**Authors:** Alicia Ackerman, Halalisiwe Khanyi, Seelan Pillay

**Affiliations:** 1 Discipline of Emergency Medicine, University of KwaZulu-Natal, Durban, South Africa

**Keywords:** emergency medicine, procedural sedation and analgesia, trauma care, orthopaedic manipulations, acute burn care

## Abstract

**Background:**

Procedural sedation and analgesia (PSA) is a core competency of emergency medicine physicians. Although international guidelines and research have advanced considerably, limited data exist on PSA practice in South African emergency departments, particularly within KwaZulu-Natal.

**Methods:**

We conducted a retrospective review of documented PSA practice at Dr Pixley Ka Isaka Seme Memorial Hospital (DPKISMH) between 01 June 2024 and 01 June 2025. Demographic data, clinical indications, preprocedural preparation, monitoring standards, drug class use, and complications were extracted from procedural sedation forms and patient records. Descriptive statistics were applied to summarise findings.

**Results:**

A total of 165 documented cases of PSA were analysed. Orthopaedic manipulations accounted for the majority of procedures (69.7%), followed by burn wound care (16.3%) and intercostal drain placement (7.9%). Patients were predominantly young adults without comorbidities, though the cohort spanned a broad age range from 18 months to 87 years. Preprocedural preparation revealed variable fasting status, with supplemental oxygen applied in most cases. Ketamine was the predominant agent, frequently supplemented with opioids for analgesia. Monitoring adhered to the Emergency Medicine Society of South Africa (EMSSA) recommendations, with most cases documented as conducted under a three-provider model. Documented complications were mild and transient, with no aspiration or vomiting reported in this cohort.

**Conclusion:**

This study reviewed documented PSA practices in a regional emergency department (ED) in KwaZulu-Natal, describing patient demographics, clinical indications, preprocedural preparation and monitoring standards, drug class use, and complication frequency.

**Contribution:**

The findings provide a baseline for understanding current practice and highlight areas for further development and research across South African emergency departments.

## Introduction

Procedural sedation and analgesia (PSA) is emerging globally as a cornerstone of emergency medicine, enabling clinicians to perform painful, distressing, or technically demanding procedures while maintaining patient safety and comfort.^1,2^ Procedural sedation and analgesia facilitate timely interventions in the emergency department (ED), circumventing delays and resource demands associated with theatre-based anaesthesia.^3^ International literature has consistently affirmed the safety and efficacy of PSA when performed with informed drug agent selection, adherence to monitoring protocols, and with clinicians who are appropriately trained.^4^

Emergency departments in South Africa often face intense pressure from high patient volumes, limited resources, and heavy trauma caseloads. In this setting, the ability to deliver safe and effective PSA is both a clinical necessity and a strategic imperative.^5^ Recognising this, the Emergency Medicine Society of South Africa (EMSSA) has formally identified PSA as a core competency for emergency medicine practitioners and, in 2020, issued comprehensive guidelines to support evidence-based practice in EDs.^2^ These guidelines outline recommended agents, monitoring standards, team roles, and documentation requirements, and they align with international standards such as those of the American College of Emergency Physicians and the South African Society of Anaesthesiologists.^6,7^ Together, they establish clear expectations for safe and effective PSA delivery across the country.

However, guideline publication alone does not guarantee uniform implementation. Real-world adherence is shaped by local workflows, clinician experience, equipment availability, and institutional culture, all of which vary considerably across regions.^8^ Clinical audits conducted in Cape Town and Gauteng have demonstrated that PSA is often inconsistently delivered, with inconsistencies around documentation, fasting status, and monitoring standards.^9,10,11^ These findings underscore a gap between formal recommendations and everyday practice, suggesting that while PSA is a frequently used tool in emergency medicine, its application remains variable. Such variation is not unique to South Africa; international literature similarly notes that adherence to PSA guidelines is influenced by local context, resource constraints, and workforce training.^12^ Examining PSA variation in resource-limited emergency settings such as this one, with a high trauma burden, could potentially broaden understanding of its use and extend its practical boundaries in further research.^13^

Published South African studies have strengthened the evidence base in the Western Cape and Gauteng, including district hospital audits of safety and efficacy, as well as descriptive accounts of paediatric PSA in Mitchells Plain.^11,14^ These contributions have provided valuable insights into how PSA is delivered in urban centres and have highlighted both strengths and areas for improvement. Nevertheless, KwaZulu-Natal remains an uncharted frontline in the national evidence landscape. Despite being a province with both urban and rural populations and one of the highest trauma burdens in the country, PSA practice in KwaZulu-Natal emergency departments has not been systematically described.^15^ This absence represents a critical evidence gap; without descriptive accounts from the region, it is unclear how PSA is delivered, whether monitoring standards are consistently applied, and how practice aligns with national and international experience.

This study was therefore designed to provide a descriptive account of PSA practice in a regional ED in KwaZulu-Natal. Its objectives were to classify the patient population undergoing PSA, identify the indications for its use, outline pre-procedural preparation and planning, assess the monitoring standards applied during the procedure, document the drug classes utilised, and report the documented frequency of PSA-related complications. This study fills a critical gap in knowledge and offers a clearer picture of how PSA is delivered in a province that has previously been absent from published data. In doing so, it strengthens the national evidence base, highlights areas for potential improvement, and provides a foundation for future work aimed at optimising PSA practice across South African emergency departments.

## Research methods and design

### Research setting

This study was carried out at Dr Pixley Ka-Isaka Seme Memorial Hospital (DPKISMH), a Regional Hospital located in KwaMashu, KwaZulu-Natal, South Africa.^16^ The hospital lies approximately 25 km south of King Shaka International Airport, positioning it within the northern Durban metropolitan corridor. As a regional hospital, it is a 500-bed unit and provides specialist services while supporting two district hospitals, four community health centres, and their associated satellite primary health care clinics.^17^ The ED at DPKISMH is one of six departments in the province led by Emergency Medicine specialists, making it a key referral centre within the KwaZulu-Natal health system.

### Study design and location

This was a retrospective, descriptive, observational study of documented PSA practice. Records of patients who underwent PSA in the ED at DPKISMH between 01 June 2024 and 01 June 2025 were reviewed for inclusion.

### Study population and sampling strategy

A convenience sampling strategy was utilised, including all documented patients who required PSA in the ED at DPKISMH between 01 June 2024 and 01 June 2025.

### Sample size

Because this was a descriptive study, no formal power calculation was undertaken, and a definitive sample size was not set in advance. Instead, the target sample size was guided by the scope and design of comparable international studies of procedural sedation, which typically report cohorts of 100–200 patients.^10,14,18^ Based on this precedent and feasibility in our setting, a pragmatic target of approximately 150 cases was chosen. This number was intended to provide a sufficiently broad dataset for descriptive analysis while remaining achievable within the available timeframe and resources.

### Inclusion criteria

All documented patients who required PSA to be performed in the ED from 01 June 2024 to 01 June 2025.

### Exclusion criteria

- Documented PSA performed before or after the study period.
- Documented PSA performed at locations outside the emergency department.
- Documented patient age < 18 months, as PSA is contraindicated for use in this population^2^.
- The documented indications for the use of sedative and/or analgesic drug agents were not for PSA.

### Data collection methods and procedures

Patients who required PSA in the ED at DPKISMH between 01 June 2024 and 01 June 2025 were identified by their treating doctors. Each case was documented, as per institutional protocol, on a prepopulated unit-specific procedural sedation form and filed in the patient’s hospital folder. At the beginning of the study period, the principal investigator cross-checked the ED resuscitation drug register to locate hospital files in which PSA related medications had been administered. These drugs included those available in the selected ED: Ketamine, Propofol, Etomidate, Morphine, Pethidine, Fentanyl, Diazepam, Midazolam, Ativan, Flumazenil, and Naloxone. With regards to the opioids, the folders were extracted for patients who had received both an opioid and a sedative during their ED stay, to meet the true definition of PSA.^19^ These sedation forms, as well as patient folders, served as the source documents. Records were reviewed against the study’s inclusion and exclusion criteria, and those meeting eligibility were enrolled. Any discrepancies were discussed with the supervisor and co-supervisor, and a consensus on inclusion or exclusion was reached in collaboration. Study data were then extracted from the identified source documents using a paper-based data collection tool. The data collection tool did not contain any identifying data to ensure anonymity.

These paper records were then transcribed into electronic format using Microsoft® Excel® (Redmond, Washington, United States). The electronic dataset was analysed, summarised, and presented as graphs, charts, and tables, with descriptive statistics used for quantitative reporting. All electronic data were stored on a password-protected laptop with backup to a secure, password-protected online server. The paper-based tools were kept in a lever arch file, locked in an access-controlled office.

### Data analysis

Descriptive statistics were employed for this study. The collected data were summarised and quantitatively described with assistance from the University of KwaZulu-Natal Biostatistics Department.

### Ethical considerations

For this retrospective clinical chart review, data collection measures were in place to ensure confidentiality and anonymity of information collected. The University of KwaZulu-Natal, Biomedical Research Ethics Committee (BREC/00008689/2025), Department of Health (National Health Research Database [NHRD] Ref: KZ_202506_012), and hospital approval (DPK07/25) were obtained prior to research commencement. Due to the retrospective nature of the study, a waiver of informed consent was requested from the BREC committee.

## Results

**FIGURE 1:**
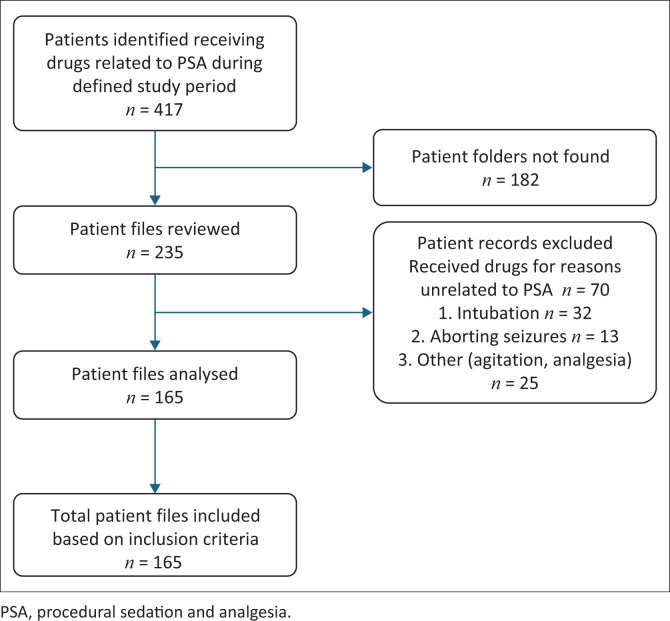
Patient selection.

In total, 417 records were reviewed: 182 unavailable, 165 included, and 70 excluded as non-PSA cases (Figure 1).

### Patient demographics

During the study period, 165 patients underwent PSA in the emergency department (Table 1). The cohort was predominantly young trauma patients, though cases spanned from early childhood (18 months) to advanced age (87 years). Most patients had no documented comorbidities. Of those files with documented comorbidities, HIV and hypertension were the most frequent. Orthopaedic procedures were the leading indication, followed by burn wound care, with fewer cases of intercostal drain placement, cardioversion, and minor surgical interventions.

**TABLE 1:** Demographics and indications for patients undergoing procedural sedation and analgesia (*N* = 165).

Patient demographics	Total (*n* = 165)	Range (Min–Max)	Mean	s.d.	% of total
**Age (years)**	-	1.5–87	38.07	18.08	-
**Male**	102	-	-	-	61.8
**Female**	63	-	-	-	38.2
**Medical comorbidities**					
HIV	22	-	-	-	13.3
Hypertension	12	-	-	-	7.2
Diabetes	5	-	-	-	3.0
Pregnancy	1	-	-	-	0.6
Heart disease	2	-	-	-	1.2
Active tuberculosis	0	-	-	-	0.0
Epilepsy	4	-	-	-	2.0
Obstructive lung disease	2	-	-	-	1.2
Not specified	10	-	-	-	6.1
**Indications**		-	-	-	
Fracture manipulation and/or joint reduction	115	-	-	-	69.7
Burns scrub	27	-	-	-	16.3
Electrical cardioversion	1	-	-	-	0.6
Abscess drainage	1	-	-	-	0.6
ICD placement	13	-	-	-	7.9
Extensive suturing	2	-	-	-	1.2
Failed local/regional nerve block	2	-	-	-	1.2
Advanced imaging	0	-	-	-	0.0
Foreign body removal	0	-	-	-	0.0
Other (hernia reduction)	4	-	-	-	2.4

PSA, procedural sedation and analgesia; s.d., standard deviation; HIV, human immunodeficiency virus; ICD, intercostal drain.

### Preprocedural preparation and monitoring standards

Consent for PSA was generally documented, most often verbally (Table 2). Fasting status was inconsistently recorded, with both fasted and non-fasted patients represented. Supplemental oxygen was commonly administered, typically via face mask, though documentation of the delivery method was sometimes incomplete. Procedural sedation and analgesia were most frequently conducted by three-person teams, with two-person and occasional single-clinician approaches also observed. Of note, the single-clinician approach is based on the assumption that a nursing assistant is present, as per the minimum monitoring standards. Monitoring during PSA was generally comprehensive, with most patients receiving electrocardiogram (ECG), pulse oximetry, and non-invasive blood pressure assessment. Minimum 5-min monitoring intervals, as recommended by EMSSA guidelines, were achieved in a subset of cases. Baseline vital signs were consistently documented, while discharge vitals were recorded in the majority. Discharge vital signs were recorded when the procedure was complete, and the patient was deemed fit for discharge, meeting predefined discharge criteria available as a checklist on the PSA forms utilised (returned to baseline Glasgow Coma Scale [GCS], vital signs within pre-sedation range, normal saturations off oxygen, no ongoing symptomatology, pain and nausea controlled).

**TABLE 2:** Preprocedural preparation and monitoring standards (*N* = 165).

PSA preparation and monitoring	Total (*n* = 165)	% of total
**Consent**		
Verbal	74	44.8
Written	32	19.3
Not specified	59	35.8
**Fasting status**		
Fasted (Last meal > 4 h, clear fluid > 2 h)	53	32.1
Not fasted (Last meal < 4 h, clear fluid < 2 h)	27	16.3
Not specified	85	51.5
**Supplemental oxygen**		
No supplemental oxygen applied	52	31.5
NPO_2_	10	6.1
40% facemask	57	34.5
NRB	13	7.9
Not specified	33	20.0
**Number of providers**		
Single provider	9	5.5
Two providers	61	37.0
Three providers	93	56.3
Not specified	2	1.2
**Minimum monitoring standards applied**		
Sats, NIBP and ECG	150	90.9
Sats and NIBP	7	4.2
ETCO_2_	0	0.0
Not specified	8	4.8
**Monitoring standards during the procedure**		
Baseline vital signs:		
Y	152	92.1
N	13	7.9
5-min interval vital signs recorded:		
Y	43	26.1
N	122	73.9
Discharge vital signs post-procedure:		
Y	141	85.5
N	24	14.5

NPO_2_, nasal prong oxygen; NRB, non-rebreather; Sats, Saturations; NIBP, non-invasive blood pressure; ECG, electrocardiogram; ETCO_2_, end-tidal carbon dioxide; Y, yes; N, no; PSA, procedural sedation and analgesia.

### Procedural sedation and analgesia personnel

In this study, PSA was most often delivered using a three-provider model comprising a sedationist, a proceduralist, and an assistant (Table 3). Medical officers with a diploma in primary emergency care typically served as sedationists, other medical officers as proceduralists, and registered nurses most frequently as assistants.

**TABLE 3:** Description of skill level of procedural sedation and analgesia personnel.

Personnel	Specialist	Registrar	MO (DipPEC[SA])	MO	Intern	RN
PSA provider (sedationist)	1	13	71	74	3	-
Proceduralist	-	9	21	60	15	-
Assistant	2	1	5	1	5	127

MO (DipPEC[SA]), medical officer with a diploma in primary emergency care, South Africa; MO, medical officer; RN, registered nurse; PSA, procedural sedation and analgesia.

### Drug agents utilised in procedural sedation and analgesia

**FIGURE 2:**
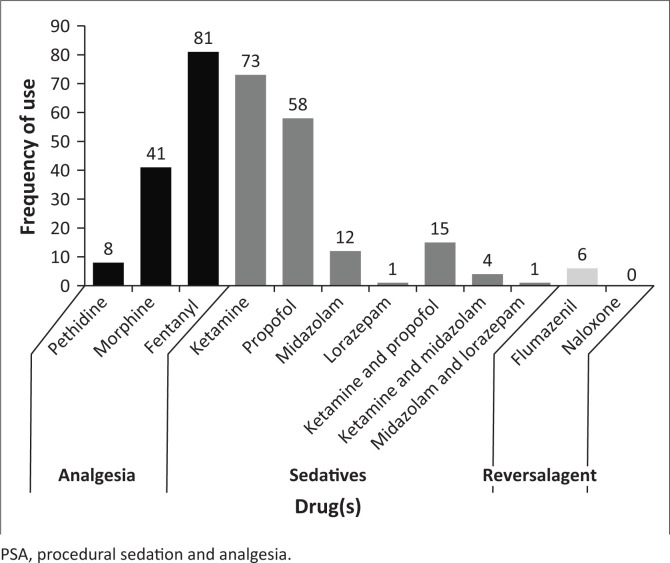
Drug classes utilised for procedural sedation and analgesia.

Analgesia was administered in most cases, with fentanyl as the predominant opioid, followed by morphine and occasional use of pethidine. Sedative agents included ketamine, classified as a dissociative sedative at PSA doses, and propofol, with benzodiazepines used less frequently. Combination regimens, most often ketamine with propofol, were employed in a minority of cases. Reversal was required in a small number of cases, all of which involved midazolam (Figure 2).

### Frequency of documented complications

Adverse events were uncommon and mild, with an overall frequency of 13.3% (Table 4). Hypoxia, bradycardia, hypotension, and emergence reactions were observed infrequently, while sedation and procedure failure accounted for most complications. No aspiration or vomiting events were documented.

**TABLE 4:** Frequency of documented complications.

Adverse event	AE frequency and drug combination associated (*n* = 165)			AE frequency–special population-elderly or paediatric (*n* = 23)	AE frequency–supplemental oxygen applied (*n* = 80)
	*n*	%	Drug(s) recorded		
Hypoxia (Sats < 90%)	4	2.4	-	0	3
	2	-	Fentanyl + Propofol	0	3
	2	-	Fentanyl + Ketamine	0	0
Bradycardia (HR < 50)	3	1.8	-	0	2
	2	-	Morphine + Propofol	0	1
	1	-	Ketamine + Propofol	0	1
Emergence reactions	4	2.4	-	0	1
	2	-	Morphine + Ketamine	0	0
	1	-	Propofol + Ketamine	0	1
	1	-	Ketamine	0	0
Hypotension (SBP < 90)	2	1.21	-	0	1
	1	-	Midazolam + Ketamine	0	1
	1	-	Fentanyl + Ketamine	0	0
Sedation or procedure failure	9	5.45	-	2	4
	3	-	Ketamine	2	1
	1	-	Fentanyl + Lorazepam	0	1
	1	-	Morphine + Propofol	0	1
	2	-	Pethidine + Midazolam	0	0
	1	-	Fentanyl + Ketamine	0	1
	1	-	Midazolam	0	0
Vomiting or aspiration	0	0.0	-	0	0
	-	-	-	0	0
Total (*n* = 165)	22	13.3	-	-	-

Sats, saturations; SBP, systolic blood pressure; HR, heart rate; AE, adverse events.

## Discussion

The demographic profile of PSA patients in this study underscores the substantial trauma burden in KwaZulu-Natal, with a mean age of 38.07 years and a predominance of otherwise healthy young men.

The youngest patient was 18 months, aligning with EMSSA guidance that children younger than this require specialist support. In older children, fasting principles remain common practice, though their benefits are disputed. Stewart et al. showed that strict fasting guidelines did not reduce adverse events, delays in care, or prolonged ED stays.^20^ These findings were echoed locally in Cape Town by Dunn et al. and reinforced internationally by Green et al., who released a consensus that now recognises that fasting does not ensure an empty stomach, and vomiting during PSA rarely results in clinically significant aspiration.^14,21^ Our cohort similarly had no documented aspiration or vomiting events.

At the opposite end of the age spectrum, PSA was utilised in this cohort up to 87 years of age, including 17 patients > 65 years of age. There is no documented maximum age that precludes PSA utilisation; however, the American College of Emergency Physicians (ACEP) and EMSSA identify elderly patients (> 65 years) as a special risk group requiring careful titration of drugs, consideration of cardio-stable alternatives, and enhanced monitoring.^2,7^ Yuan et al. further highlighted the hemodynamic concerns of propofol in older adults, supporting the use of more stable drug classes in this population.^22^ Of note, no cases of hypotension were recorded among the elderly population in our study. An American Society of Anesthesiologists (ASA) grading would have been particularly beneficial in the elderly in determining risk of PSA and guiding drug choices, which was not within the scope of this study.

Against the background of KwaZulu-Natal’s high trauma burden, our study found that orthopaedic manipulations were the leading indication for PSA, followed by burn wound scrubbing and intercostal drain placement. Local studies by Wood Thompson et al. report a similarly trauma-dominant profile; however, in contrast, cardioversion was the most frequent indication in their cohort, with fracture reductions, paediatric suturing, and advanced imaging in uncooperative children following closely.^5^ The absence of a dedicated paediatric trauma and medical unit at DPKISMH, a regional referral facility, likely accounts for the limited use of PSA for advanced imaging in paediatric patients. In line with the established referral pathway, cases requiring advanced imaging and regional care are managed at other facilities in the KwaZulu-Natal region.

Internationally, Smit et al. in the Netherlands identified orthopaedic manipulations, abscess drainage, and cardioversion as frequent indications, whereas Sacchetti et al. in the United States highlighted orthopaedic procedures, lumbar puncture, and advanced imaging as their most common indications.^23,24^ These variations across centres underscore the difficulty of standardising PSA practice and highlight the need for comprehensive guidelines to ensure safe, effective care across the broad spectrum of indications we encounter.

Within the given context of KwaZulu-Natal’s high trauma burden, as noted, orthopaedic reductions were found to be the leading indication for PSA in our study. Kuypers et al. compared PSA with regional anaesthesia for fracture manipulations, finding similar patient satisfaction, fewer adverse events, and shorter ED stays.^25^ Regional anaesthesia therefore offers a pragmatic alternative in resource-constrained settings, either as a stand-alone technique or as an adjunct to PSA for enhanced pain control and earlier discharge. South African data reveal strong clinician interest in peripheral nerve blocks but limited formal training, emphasising the need for structured educational initiatives to support wider adoption.^26^

Iorember et al. identified informal housing, unsafe cooking fuels, and socioeconomic inequality as key drivers of burn injuries in African settings.^27^ Given these determinants and with our study conducted in a catchment area that includes communities facing socioeconomic challenges, it is unsurprising that burns emerged as the second most common indication for PSA.

The Emergency Medicine Society of South Africa recommends that PSA in the ED be performed by a minimum of two trained clinicians: one dedicated to performing the procedure and a seditionist, who is solely responsible for monitoring the patient after administering the appropriate drugs.^2^ This dual provider model ensures that patient safety is not compromised while the operator focuses on the intervention. This study found that the majority of the PSA conducted was aligned with this recommendation. There were nine documented cases of single-provider PSA in this cohort, with assistance from an RN rather than a dedicated clinician. Although not ideal, Vinson et al. demonstrated that PSA conducted by a single physician with support from a nurse trained in sedation was sufficient, with no documented complications.^28^ Similarly, Josephy et al. found that in appropriate settings, the single-provider model, namely a physician supported by a sedation-trained nursing member, was feasible and safe, with no increase in adverse events (Figure 2).^29^ Together, these studies suggest that single-provider PSA with trained nursing support can be delivered safely under defined conditions, an important consideration in this setting.

Routine oxygen administration during PSA is recommended by current guidelines; however, recent evidence challenges this approach. Fu et al. showed that supplemental oxygen can impair detection of hypoventilation by pulse oximetry, as saturations may remain reassuring while carbon dioxide accumulates silently, masking apnoea.^30^ Saunders et al. further suggest that with vigilant monitoring, including pulse oximetry and capnography, oxygen may be reserved for patients with respiratory compromise, anticipated airway difficulty, or prolonged sedation.^31^ Of note, in our study, three out of the four patients who developed hypoxia had received routine oxygen, underscoring the risk of delayed recognition and the value of capnography for early detection.^32^ Clinical judgement, supported by ventilatory monitoring, should guide oxygen use rather than rigid protocol mandates. Unfortunately, capnography was not available in this study setting.

Documented analgesic choices in this study reflected departmental culture, with fentanyl and morphine being the most frequently administered agents. In this cohort, ketamine was classified as a sedative agent as, at higher PSA doses, its predominant effect is dissociation accompanied by amnesia and sedation, with analgesia considered a secondary role at lower doses.^6^ It was the most frequently used drug in our cohort, followed by propofol. Unfortunately, the exact dose could not be determined as the patient’s weight was not routinely measured. A smaller proportion of patients received ketamine and propofol combinations, though titration strategies were not explored. These findings mirror local PSA practices, with Meyer et al. reporting similar drug use at Steve Biko Academic Hospital in Johannesburg.^11^ In contrast, Hodkinson et al. documented morphine and midazolam as the most common agents in a Cape Town cohort, yet noted that nearly half of clinicians (47.4%) would personally prefer propofol if they themselves required PSA, raising questions about the perceived effectiveness of morphine and midazolam combinations.^33^ Of note, reversal agents were administered in six cases, specifically flumazenil following midazolam use. This practice diverges from recent American Heart Association guidance, which cautions that benzodiazepine reversal may precipitate seizures and dysrhythmias.^34^ They recommend its use only in narrowly defined circumstances such as severe iatrogenic benzodiazepine overdose with impending arrest, a complication that was not documented in any case in this study.

A critical lesson is that PSA must never be approached with a ‘one drug fits all’ mentality; the choice and dose of agents must be tailored to the individual patient and the specific procedure, as uniform regimens risk compromising both safety and efficacy.

In our cohort, the complications observed were predominantly mild, with no documented cases requiring immediate invasive intervention. An overall complication rate of 13.3% was recorded, though many events were attributable to procedural or sedation-related failures. As these categories are not routinely considered complications in systematic reviews such as that by Bellolio et al., exclusion yielded an adjusted rate of 7.9%, which is relatively consistent with their reported average of 2% – 5%.^35^ Importantly, no episodes of aspiration or vomiting were found to be documented, despite only a third of the patients being reportedly fasted. Of interest, both recorded cases of adverse events involving the elderly or paediatric populations were related to Ketamine, and both were procedure and/or sedation failures, possibly reflecting dosing concerns relating to weight and pharmacokinetics in these populations. While these observations are consistent with reports that serious adverse events are uncommon and that most complications are transient (e.g. hypoxia or hypotension), our findings should be interpreted cautiously.^36^ They reflect what was documented in available records and do not permit definitive conclusions regarding the overall safety profile of PSA.

This study provides key insights into real-world PSA practice at DPKISMH. Strengths of this study include its being conducted at one of the six specialist-driven EDs in KwaZulu-Natal within an academic facility where emergency medicine registrars rotate. Additional strengths include the use of a structured PSA form for every sedation, enabling more reliable data capture and adherence to EMSSA recommendations. The inclusion of all PSA cases over a full year enhanced the sample’s representativeness, given the anticipated seasonal variation in PSA indications. Limitations include the retrospective design and documentation gaps that we were unable to clarify. The data available were dependent on the quality and completeness of the clinical documentation available, potentially leading to selection bias. The utilisation of the drug book could have potentially led to cases being missed if improper documentation was carried out. Further constraints arose from the inability to locate a significant number of patient folders. The absence of documentation of ASA classification was a further limitation.

## Conclusion

We aimed to evaluate documented PSA practice in a regional ED in KwaZulu-Natal. The study classified the patient population, demonstrating a predominance of young, otherwise healthy trauma patients, but extended across the age spectrum, including paediatric and elderly groups. Documented indications were dominated by orthopaedic manipulations, followed by burn wound care and intercostal drain placement, reflecting the trauma burden in the catchment area. Review of preprocedural preparation found that fasting status was variable amongst the cohort, with the utilisation of supplemental oxygen noted as standard practice. Documented monitoring standards largely adhered to EMSSA recommendations, with most cases reportedly conducted under a three-provider model. Drug use patterns revealed ketamine as the predominant agent for procedural sedation, classified as a sedative due to its dominant dissociative and amnestic effects at PSA doses. Its intrinsic analgesic properties were supplemented by opioids, most commonly morphine, to provide additional analgesia. Documented complications were mild and transient, with no episodes of aspiration or vomiting. These findings provide insight into PSA practice within a specialist-driven ED in KwaZulu-Natal, while highlighting opportunities to enhance record keeping, expand training in regional nerve blocks, and pursue multicentre research to capture practice variation across South African emergency departments.
